# Synthetic protein protease sensor platform

**DOI:** 10.3389/fbioe.2024.1347953

**Published:** 2024-04-05

**Authors:** Ciaran Devoy, Yensi Flores Bueso, Stephen Buckley, Sidney Walker, Mark Tangney

**Affiliations:** ^1^ Cancer Research@UCC, University College Cork, Cork, Ireland; ^2^ APC Microbiome Ireland, University College Cork, Cork, Ireland; ^3^ IEd Hub, University College Cork, Cork, Ireland

**Keywords:** synthetic biology, protease activity, Mpro protease, sandwich ELISA, diagnostic, MMP9 and TEV

## Abstract

**Introduction:** Protease activity can serve as a highly specific biomarker for application in health, biotech, and beyond. The aim of this study was to develop a protease cleavable synthetic protein platform to detect protease activity in a rapid cell-free setting.

**Methods:** The protease sensor is modular, with orthogonal peptide tags at the N and C terminal ends, which can be uncoupled via a protease responsive module located in between. The sensor design allows for several different readouts of cleavage signal. A protein ’backbone‘ [Green fluorescent protein (GFP)] was designed *in silico* to have both a C-terminal Flag-tag and N-Terminal 6x histidine tag (HIS) for antibody detection. A protease cleavage site, which can be adapted for any known protease cleavage sequence, enables the uncoupling of the peptide tags. Three different proteases—Tobacco, Etch Virus (TEV), the main protease from coronavirus SARS-COV-2 (Mpro) and Matrix Metallopeptidase 9 (MMP9)—a cancer-selective human protease—were examined. A sandwich Enzyme-Linked Immunosorbent Assay (ELISA) was developed based on antibodies against the HIS and Flag tags. As an alternative readout, a C-terminal quencher peptide separable by protease cleavage from the GFP was also included. Purified proteins were deployed in cell-free cleavage assays with their respective protease. Western blots, fluorescence assays and immunoassay were performed on samples.

**Results:** Following the design, build and validation of protein constructs, specific protease cleavage was initially demonstrated by Western blot. The novel ELISA proved to afford highly sensitive detection of protease activity in all cases. By way of alternative readout, activation of fluorescence signal upon protease cleavage was also demonstrated but did not match the sensitivity provided by the ELISA method.

**Discussion:** This platform, comprising a protease-responsive synthetic protein device and accompanying readout, is suitable for future deployment in a rapid, low-cost, lateral flow setting. The modular protein device can readily accommodate any desired protease-response module (target protease cleavage site). This study validates the concept with three disparate proteases and applications–human infectious disease, cancer and agricultural crop infection.

## 1 Introduction

Synthetic protein design and technology is a rapidly evolving field (Walker et al., 2021). As the technology progresses, opportunities increase for exploitation of designer proteins in detecting targets of interest (Flores Bueso and Tangney, 2017). More traditional proteins (antibodies) are already commonplace as part of rapid ‘in the field’ tests, such as lateral flow tests used for the detection of biomarkers including microbial antigens (e.g., COVID-19 antigen tests) (Candel et al., 2020) or hormones (pregnancy tests) (Leuvering et al., 1980). Novel strategies, however, need not be restricted to the immune-binding of targets, with the potential to design proteins as ‘sensors’ of biological or chemical activity.

Proteases can act as highly specific indicators of the activity of multiple biological processes and can therefore serve as useful as biomarkers for a wide range of targets of value in health, food and other key sectors of global social and economic importance. Every living organism has associated proteases involved in functions such as digestion, waste degradation, cell signalling and post-translational modification. These processes are usually highly regulated, with dysregulation being an indicator of disease. The human genome has over 600 proteases, and these are involved in virtually every stage of a cell’s life cycle (Bond, 2019). Proteases associated with human disease range from dysregulated endogenous proteases involved in cancer progression or inflammatory disease to prokaryotic and viral pathogens. The majority of human disease causing viruses encode at least one protease (Sharma and Gupta, 2017). The ability to rapidly detect and amplify this signal of protein cleavage could prove invaluable in a diagnostic setting for a wide array of human diseases.

Proteases hydrolyse peptide bonds and can be divided into six classes based on the mechanism of action. Aspartic, glutamic and metalloproteases use an activated water molecule as the nucleophile to attack the peptide bond. In contrast, with cysteine, serine, and threonine proteases, the nucleophile is the amino acid residue, after which they are named (López-Otín and Bond, 2008). The substrate specificity is based on protein/protein interactions between the substrate and protease in the binding pocket of the enzyme, with substrate side chains accommodated in side pockets (Schauperl et al., 2015). Typically, proteases recognise six to eight amino acid residues with varying levels of specificity ranging from the most stringent with single peptide bond recognition in the case of angiotensin-converting enzyme to proteinase K, which is broad spectrum (Zhou et al., 2020). Knowing the cleavage site amino acid sequence of any given protease means it can be engineered as part of a synthetic protein. Cleavage site sequence information can be found on the MEROPS database (Rawlings and Bateman, 2021). The proteases chosen for this study have substrates that do not overlap (although cleavage by proteases with overlapping substrate specificity cannot be ruled out completely (Schauperl et al., 2015)).

Currently, protease activity can be detected using laboratory instruments using a number of methods, reviewed elsewhere (Ong and Yang, 2017; Chung and Lin, 2020). Briefly, these include mass spectrometry-based assays (Yan et al., 2011; Ritorto et al., 2014); colourimetry-based chemistry (Guarise et al., 2006; Lou et al., 2010); fluorescent protein-based techniques (Heim et al., 1994; Zhang, 2004; Callahan et al., 2010; Nicholls et al., 2011; Stawarski et al., 2014); bioluminescence based enzymes (Yao et al., 2007; Wigdal et al., 2008); quantum dots (QDs) (Medintz et al., 2006); nanoparticles (Berdichevsky et al., 2003; Laromaine et al., 2007; Jin et al., 2012; Song et al., 2013; Loynachan et al., 2019). More recent developments involve using liquid crystal (LC) based protease assays which can allow for naked-eye detection of protease activity (Jannat and Yang, 2018). Transcription-based assays have also been described and offer extremely high sensitivity (Liu et al., 2021; Fink and Jerala, 2022) In terms of existing diagnostic kits, these can be divided into two broad categories: nucleic acid-based molecular diagnosis and antigen/antibody-based immunoassays. Nucleic acid quantification methods include quantitative reverse transcription-polymerase chain reaction (qRT-PCR), loop-mediated isothermal amplification (LAMP) based assays, clustered regularly interspaced short palindromic repeats (CRISPR)-associated protein (Cas) system, and RNA sequencing, with qRT-PCR currently deemed the gold standard for COVID-19 detection. For a test which could be used at home, a lateral flow assay (LFA) which works on the same principle as the sandwich ELISA presented in this study, could be adapted quite easily using the same antibodies (Yalow and Berson, 1960). Pregnancy tests, and more recently, the COVID-19 pandemic, have brought LFAs to the fore of at-home tests worldwide.

**FIGURE 1:**
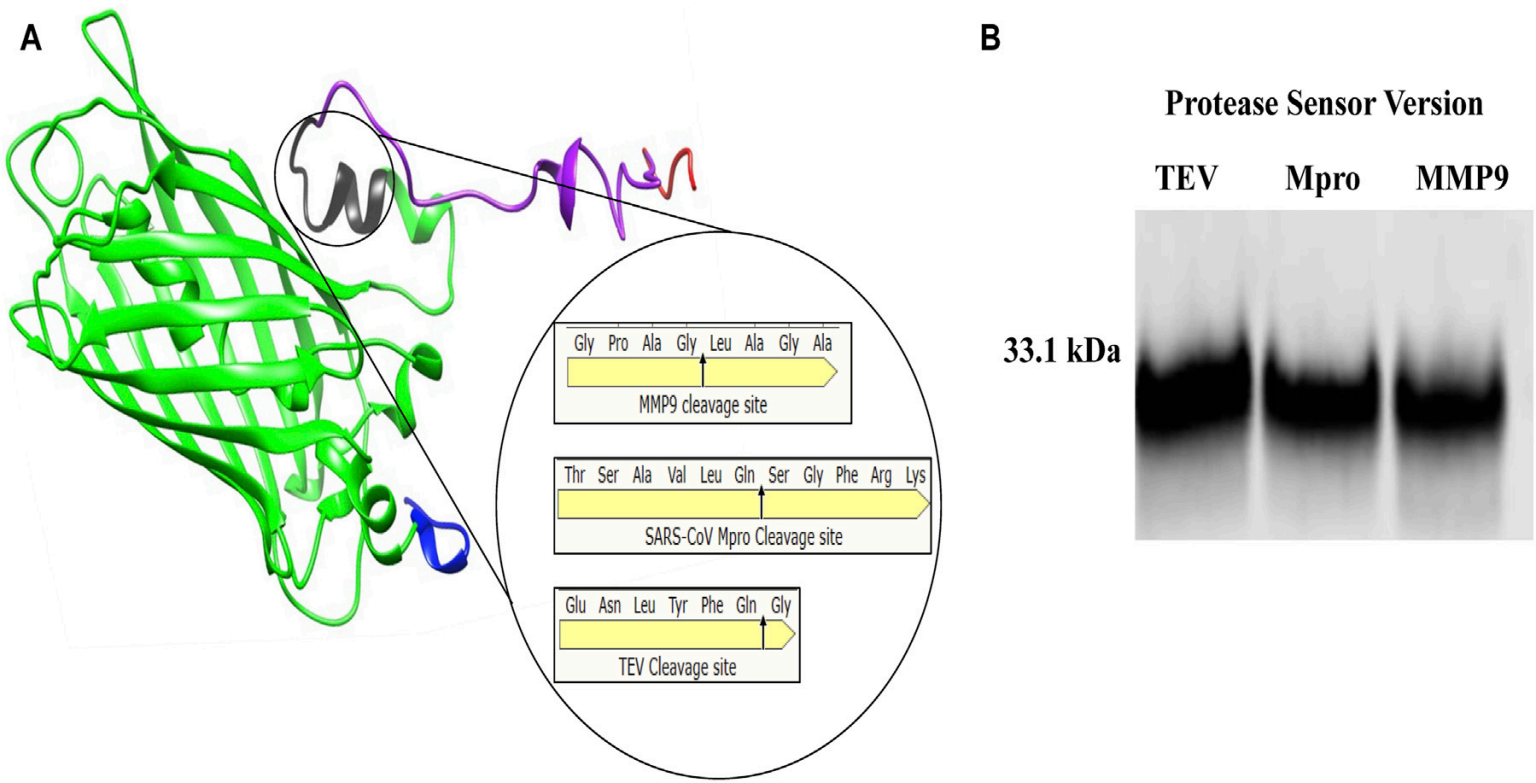
*Protease sensor Design* **(A)** *3D model of engineered protease cleavable synthetic protease sensor (33.1 kDa), showing Flag-tagged N-terminus (blue), 27 kDa GFP beta-barrel (green) with cleavage site in (black), M2 quencher (purple) and HIS tagged C terminus (red) Magnified cleavage site with amino acid cleavage sequence for each of the three chosen proteases (TEV, MMP9 and Mpro)* **(B)** *Western blot α-Flag showing each of the 33.1 kDa protease sensor versions TEV, Mpro and MMP9.*

This study describes the design, build and test of a novel protein-based platform for potential use as a rapid test for any protease, comprising a synthetic protein with a sensor, readout modules and accompanying readout. A quenched GFP was chosen as the backbone of the sensor, allowing for confirmation of cleavage through fluorescent signal detection. The incorporation of opposing distinct peptide tags allowed for protease activity to be detected by sandwich ELISA using well characterised commercially available antibodies, significantly increasing its sensitivity. Three unrelated protease cleavage sequences were chosen to validate this approach’s versatility - TEV, MMP9, and Mpro. Tobacco, Etch Virus (TEV) is one of the most routinely used proteases for in research and industry (Kapust et al., 2001). Mpro is the viral protease from the SarsCov-2 virus (Jin et al., 2020), and MMP9 is a ubiquitous cancer-associated protease involved in cancer-mediated tissue remodelling and metastasis (Page-McCaw et al., 2007), well studied as a cancer-selective biomarker. For each protease, a protease-specific protease sensor featuring its cognate protease-specific cleavage peptide was designed*,* with only the amino acid sequence of the cleavage site varying between constructs (Figure 1A).

While there are tests for proteases that exploit ELISA, a key aim of this study is to progress the technology that is synthetic proteins as ‘smart’ tools for biotech, in particular the potential to design proteins as ‘sensors’ of biological activity. Here, we provide proof of concept for a design strategy of a synthetic protein as a sensor of biological activity, where the readout parts are ‘uncoupled’ by the target activity (protease in this case). This design has potential value in multiple settings *in vivo* or *in vitro*, for research, diagnostic or therapeutic functions. This study generates proof of concept data through validation of its use *in vitro* when coupled with an ELISA detection strategy.

## 2 Materials and methods

### 2.1 *In silico* protein design

All synthetic proteins were designed and modelled *in silico* to predict suitability for wet lab use and redesigned where necessary. The process for *in silico* modelling used was as previously described by our lab (Yallapragada et al., 2020). Briefly, *in silico* features, including Molecular Weight, Theoretical pI, Hydrophobicity (GRAVY) and Instability Index were calculated using the ProtParam facility, hosted by the Swiss Bioinformatics Resource Portal (expasy.org). The protein tertiary structures were predicted using I-TASSER suite (v5.1). This suite of tools also provides solvent accessibility estimates per residue and prediction of active sites. This additional information was combined using R to find the solvent accessibility of the active site. All generated data points were then integrated and used to rank the *in silico* designed constructs in terms of predicted performance.

A green fluorescent protein (GFP) was chosen as a protein ‘backbone’. The GFP protein chosen was GFP S65T (PDB-ID 1Q4A), is 27 KDa in size, has an S TO T mutation at the residue number 65 from wild-type *Aequorea victoria* GFP (PDB-ID 1EMA) and has excitation/emission spectra of 490 and 520 nm, respectively. A previously-described version of this protein, fused at the carboxyl-terminus to a 27 AA peptide originating from the M2 influenza proton channel was used for design (Pinto and Lamb, 2006). This peptide promotes the tetramerisation of GFP, disrupting its maturation, consequently leading to a loss of signal and is hereafter referred to as the quencher (Nicholls et al., 2011).

For the study described here, a protease cleavage site for a given protease was incorporated module between the GFP and its quencher, to permit the restoration of fluorescence following protease cleavage. A Flag-tag was added to the amino-terminus and a 6 x HIS-tag was added to the carboxyl-terminus of the protein backbone to facilitate the detection of protein cleavage.

### 2.2 DNA design

Following *in silico* validation of test sequences, the finalised constructs’ AA sequences were reverse translated into their corresponding DNA sequences using the backtranseq feature on the EBI website (https://www.ebi.ac.uk/Tools/st/emboss_backtranseq/). The DNA sequences were codon optimised for expression in *E. coli* using the codon optimisation tool available on the IDT website (https://eu.idtdna.com/codonopt). Gene Blocks were purchased from Integrated DNA Technologies (IDT, Belgium).

New England Biolabs (NEB, Ipswich, MA, USA) and SnapGene’s Gibson assembly simulators were used to design the homologous arms to facilitate Gibson assembly. Amplification and sequencing primers were designed using Primer3PLus. All primers were sourced from IDT (Belgium).

### 2.3 DNA assembly

#### 2.3.1 Conventional PCR

25 µL reactions were performed using Taq 2X Master Mix (NEB, Ipswich, MA, USA) and 0.25 µM of each primer. Cycling conditions included: an initial denaturation for 30 s at 95°C. 25–35 cycles of denaturation at 95°C for 10 s, annealing for 15 s at the primers’ optimal temperature (specified by NEB’s calculator for Taq DNA polymerase), 20–40 s of extension at 68°C (20 s for 200bp amplicons and 40 s for 400–500 bp amplicons), and a 5 min final extension at 68°C. 10 μL of amplified products were loaded to a 1.5% agarose gel, run at 180 V for 40 min, and imaged with Gel Doc EZ System (Bio-Rad, California, USA).

#### 2.3.2 Plasmids

The pRSFDuet-1 plasmid (NOVAGEN Darmstadt, Germany) was used for cloning and expression of the protease sensors. The plasmid encodes two multiple cloning sites (MCS), each preceded by a T7 promoter, lac operator, and ribosome binding site (RBS). The plasmid also carries the pRSF1030 replicon, *LacI* gene, and kanamycin resistance marker.

*Bacterial transformation: Escherichia coli* BL21 cells were made competent using Cohen et al., 1972 protocol (Cohen et al., 1972). 100 ng plasmid was mixed with 30 μL of competent *E. coli* BL21 cells and were placed on ice for 20 min. The suspension was subjected to heat shock at 42 °C for 20 min. The cells were again placed on ice for 2 min and 1 mL LB broth was added. 100 μL transformed cells were plated on LB agar containing kanamycin at 50 μg/mL. Colonies were subcultured and stored in 25% glycerol at - 80 °C for further use. For plasmid extraction, overnight subcultures of the transformed bacteria were processed through a Monarch Plasmid miniprep kit (New England Biolabs, Ipswich, MA, USA) using the manufacturer’s protocol.

#### 2.3.3 Restriction digest

Restriction enzymes used with pRSFDuet-1 were NcoI, AvrII, NdeI and BglII (NEB), as per manufacturer instructions. Following restriction digest, DNA was purified using a PCR purification kit (Qiagen, Hilden, Germany) protocol, and the digest was verified by Agarose gel electrophoresis. DNA concentration was determined using a NanoDrop spectrophotometer (Thermofisher, Waltham, MA, USA).

#### 2.3.4 Gibson assembly

Gibson Assembly was carried out using the Gibson Assembly master mix described by DG Gibson *et al.* (Gibson et al., 2009). Plasmid and DNA gene blocks were mixed in a 1:3 ratio in a Gibson Assembly master mix and incubated at 50°C. *E. coli* BL21 cells were transformed with the assembled plasmids and plated on LB agar medium with kanamycin at 50 μg/mL. Selected colonies were added to the NEB PCR master mix with 2.5 μL of corresponding primers. PCR was carried out as per NEB Q5 polymerase PCR protocol. Sanger sequencing (GATC light-run) was also performed on the selected colonies to confirm the assembly (Eurofins Genomics, UK Ltd.).

### 2.4 Protein expression and purification

#### 2.4.1 Bacterial protein production

All expression constructs described were transformed into *E. coli* strain BL21 and grown at 37 °C to an optical density (O.D) A600 of 0.6. Cultures were then induced using 1 mM isopropyl ß-D-1- thioglalctopyranoside (IPTG) and the temperature reduced to 25 °C for 3 h of expression. Cells were harvested by centrifugation at 5,000 x g for 15 min and lysed under native conditions using BugBuster (NOVAGEN Darmstadt, Germany) or under denaturing conditions using a lysis buffer containing 8 M urea, 100 mM NaH_2_PO_4_, 10 mM Tris HCl and 10 mM imidazole at pH 8.0. Clarified lysates were prepared by centrifugation at 15,000 x g for 40 min. For use as a cleaved protein size comparison, a GFP-only protein was employed (lacking the quencher module), for which the gene block was obtained from IDT (Belgium). This was purified using HIS-tag/Ni-NTA system affinity liquid chromatography. Under native conditions, the column was washed with a buffer composed of 50 mM imidazole, 300 mM NaCl, and 50 mM NaH_2_PO4 pH 8.0. Proteins were eluted using a buffer of 300 mM imidazole, 300 mM NaCl, and 50 mM NaH_2_PO_4_ pH 8.0, aliquoted and stored at–80 °C.

All protease sensors containing the M2 fluorescence quencher required purification under denaturing conditions using the following buffers; Denaturing wash buffer (wash buffer 1) - 8 M urea, 100 mM NaH_2_PO_4_, 150 mM NaCl and 20 mM imidazole at pH 8.0. Native wash buffer (wash buffer 2) - 50 mM NaH_2_PO_4,_ 500 mM NaCl and 20 mM imidazole at pH 8.0. Elution buffer 50 mM NaH_2_PO_4_, 500 mM NaCl and 250 mM imidazole at pH 8.0.

#### 2.4.2 Protein concentration, diafiltration, desalting and buffer exchange

Purified proteins were dialysed using a Slide-A-Lyser ^®^ from Thermo Scientific in a storage buffer containing 50 mM Tris and 150 mM NaCl at PH 8.0 and concentrated using an Amicon ^®^ Ultra Centrifugal Filter Devices 10K filter device, all as per manufacturer’s instructions.

### 2.5 Protein analyses

SDS-PAGE and Western blot:

An estimate of protein concentration was determined by measuring A_280__nm_ using a Thermo Scientific NanoDrop 2000 spectrometer prior to SDS page and Western blot. Concentrated proteins were solubilised in loading buffer (62.5 mM Tris-HCl pH 6.8, 2% SDS, 41.7 mM dithiothreitol, 10% glycerol, 0.01% bromphenol blue). Then boiled for 10 min at 70°C and resolved on a 4%–12% gradient SDS-PAGE gel. After running, the gel was fixed in a solution of 50% methanol and 10% acetic acid and stained with EZBlue™ gel staining reagent. The gel was imaged after staining, and subsequent washes using a Bio-Rad EZ Gel imager and analysed using the Image Lab (BioRad California, USA).

SDS-Page gels were electro-transferred to PVDF membranes for immunoblotting using Trans-Blot^©^ Turbo™ (BioRad California, USA) reagents and equipment. The membranes were blocked with 5% skim milk and incubated with indicated primary antibodies overnight at 4°C. Primary α-Flag M2 antibody was used for the detection of Flag fusion proteins (1:1,000). This mouse-raised monoclonal antibody was purchased from Merck (Darmstadt, Germany). Primary mouse monoclonal [HIS.H8] recognises HIS-tagged recombinant proteins (1:1,000). The secondary Antibody used was IRDye^®^ 800CW Goat anti-Mouse IgG from Li-Cor (1:15,000). Odyssey^®^ DLx from Li-Cor was used to image and analyse the blots. A 10–250 kDa Protein ladder Precision Plus Protein Dual Color Standards from BioRad was used to estimate protein size. The protein standard used for estimating protein concentration was an Amino-terminal FLAG-BAP™ Fusion Protein.

### 2.6 Cleavage assays

*SARS-CoV-2 Mpro*: The assay was performed with a buffer consisting of 50 mM Tris-HCl and 1 mM EDTA (pH 7.3) at 30°C for various lengths of time. 1 μg of Mpro (Merck Darmstadt, Germany) of 33.8 kDa in size with 50 µg of protease sensor 33.1 kDa.

*MMP9:* The assay was performed with a buffer consisting of 50 mm Tris/HCl, 150 mm NaCl, 10 mm CaCl_2_, 20 µM ZnCl2, 0.05% Brij35 at pH = 7.5.1 μg of MMP9 catalytic domain (Abcam, Cambridge, UK) of 40 kDa combined with 50 µg of protease sensor 33.1 kDa for various lengths of time at 37°C.

*TEV:* Carried out as per manufacturer’s instructions (NEB, Ipswich, MA, USA). 1 μL of TEV Protease per 15 μg of substrate in the provided TEV reaction buffer and incubated at 30°C for various lengths of time.

### 2.7 Fluorescence assays

Protease sensor samples containing 50 µg protein with or without associated protease were prepared at time zero to assess the ability of the protease to cleave directly. At the initiation of the reaction, 200 µL aliquots of the digest and controls were added to a Costar 96-well black plate. The fluorescence was measured at various time points (Ex. 475 nm/Em 512 nm) using a BMG FLUOstar^®^ Omega multi-mode microplate reader.

### 2.8 Enzyme-linked immunosorbent assay (ELISA)

#### 2.8.1 Antibodies, reagents, and material

Antibodies used in this study were sourced from ABCAM (Netherlands) and were previously validated for use in ELISA. These are listed in Supplementary Table S1 3,3′,5,5′-tetramethylbenzidine (TMB) substrate was acquired from Surmodics (US, Cat. No. TMBS-1000–01). All other reagents were prepared as per Supplementary Table S2 ELISAs were performed on NUNC MaxiSorp™ 96 well plates (Sigma-Aldrich, Darmstadt, DE, Cat. No. M9410).

#### 2.8.2 Standards

Standards used for these assays were purified as per protocols in Section 2.4. Proteins were verified by immunoblotting and their purity and quantity assessed via PAGE gels and spectroscopy. The full protease sensor was used as a standard to quantify uncleaved proteins and the cleaved sensor as a standard for measuring cleaved fractions. Seven-points (1/5 dilutions) standard curves, ranging from 1 to 5,000 ng of protein, were used for optimisation of the assay (antibody titration and defining sample load; Supplementary Figure S1). Six-point standard curves covering a range of 10–1,000 ng of protein (2/5 dilutions) were used for quantifying cleavage.

#### 2.8.3 Samples and plate layouts

The products of cleavage reactions with or without Mpro, TEV, or MMP9 (samples) were eluted in EB to a concentration of 1.25 ng/μL, and 125 ng (100 µL) was loaded into each well. For each protease tested, cleavage was quantified by loading a plate that included: *a)* Six replicates of each standard (full and cleaved sensor); *b)* Twelve replicates of each, matched cleaved and uncleaved reaction products, three replicates to be analysed with each primary detection antibody (α-HIS vs. α-GFP) and standard.

#### 2.8.4 Assay

*General practices:* All incubation steps, unless specified, were performed at 20°C, shaking at 350 rpm. All the solutions added to the wells were pipetted using a multichannel pipette, avoiding over-drying of the wells and were removed by swiftly inverting the plate on top of a sink and blotting against low-lint absorbent paper. All washes were performed with 300 µL of the corresponding wash solution as specified below.

*Procedure: a*) *Coating:* Wells were coated with 100 ng of Goat α-Flag in 100 µL PBS overnight at 4 °C. Unabsorbed antibody was removed from the plate and the wells were washed three times with PBS. *b*) *Blocking:* Wells were blocked with 250 µL of blocking solution for 1.5 h and the excess was removed by washing the plate three times with PBS. *c*) *Antigen binding:* Samples and standards were loaded to the respective wells and incubated for 2 h. Unbound antigens were removed from the wells with four PBS-T washes. *d*) *Antibody and conjugate labelling:* 8 ng of α-GFP or 20 ng of α-HIS antibodies in 100 µL of EB, were added to the wells and incubated for 2 h. The unbound antibody was removed with four PBS-T washes. This was followed by a 1.5 h incubation with 8 ng of α-Rabbit HRP-conjugated antibody. After which, the plates were thoroughly washed with five PBS-T washes. *e*) *Detection:* 100 µL of TMB substrate was added to the wells and the plate was incubated for 15 min in the dark and the reaction was stopped with 100 µL of sulfuric acid (1M). *e*) *Reading:* The plate was read within 5 min of adding H_2_SO_4_, with 450 nm and 570 nm absorbance filters, following the default settings of a BMC Omega plate reader. See the illustration of components in Figure 4A.

#### 2.8.5 Data interpretation and analysis

The preparation of raw ELISA data was performed with the BMG Omega–data analysis software. Here, the background was removed from the raw data by subtracting the values obtained for 570 nm absorbance. Four standard curves were plotted per plate (see Supplementary Figure S1), one per sensor (cleaved/full) detected with each antibody (α-GFP/α-HIS). These were plotted using the average of three replicates and fitted using the five-parameter logistic (5 PL) regression model to account for differences in curve symmetry. Standard curves with goodness of fit (*R* ^2^) ≥ 0.98 were deemed suitable for analysis. The resulting raw quantity of protein detected for each reaction was exported to Excel, where it was tabulated in a format compatible with R. Further analysis was performed in R using Tidyverse packages. Raw and tabulated data and R-scripts used for the analysis can be found in the Zenodo repository DOI 10.5281/zenodo.7022826. To account for differences in antibody detection (α-GFP/α-HIS) signals, the quantity of protein detected for each replicate was normalised using the uncleaved controls as normalisers. The resulting values were then plotted using ggplot2 and statistical significance was tested with Welch Two Sample t-tests.

## 3 Results

### 3.1 *In silico* design and validation of protease sensor platform

The entire 33.1 KDa construct, consisting of all the described parts (Flag + GFP + Protease cleavage signal peptide + Quencher + HIS-tag), is hereafter referred to as the protease sensor (Figure 1A). For each protease, a protease-specific protease sensor featuring its cognate protease-specific cleavage peptide was designed*,* with only the amino acid sequence of the cleavage site varying between constructs shown in magnified circle of (Figure 1A). Figure 1B is a Western blot showing the sensors of the correct size.

### 3.2 Experimental validation of protein sensors

#### 3.2.1 Specific cleavage of protease sensors as evidenced by Western blot

**FIGURE 2:**
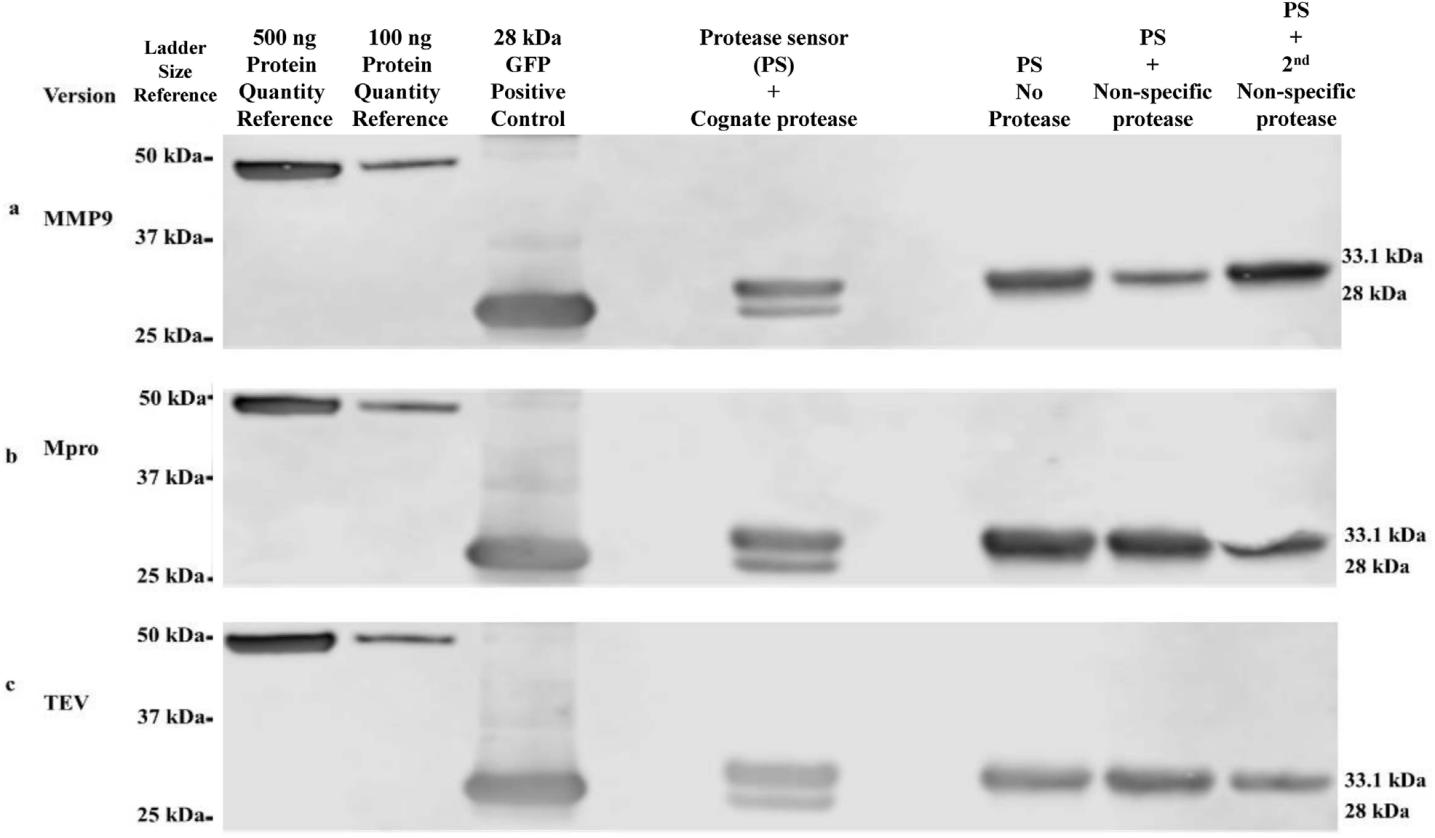
Western blot validation of protease-specific cleavage of protease sensors α-Flag antibodies were used in the detection and size determination of protease sensors ± cleavage. **(A)** MMP9 version, **(B)** Mpro version, **(C)** TEV version. Intact protease sensor corresponds to 33.1 kDa, while cleaved protease sensor (HIS-bearing terminus removed by protease cleavage) corresponds to 28 kDa.

The modular protein device can readily accommodate any desired protease-response module by incorporating the target protease cleavage site. Cleavage assays were performed on protease sensors using Western blot as readout (Figure 2). α-Flag antibody was employed to detect the cleavage assay products of the three selected proteases, along with a control for a size comparison of the cleaved product of the sensor. The cleavage of each sensor protein version was shown to be specific to its cognate protease only. Included in each cleavage assay were negative controls, including no protease/buffer only and two additional negative controls with two non-specific proteases. For example, in the case of the TEV cleavage assay, the two non-specific proteases would have been Mpro and MMP9. For each assay, cleavage was evident by densitometry at levels of 22% for TEV, 29% for Mpro and 24% for MMP9 sensors.

### 3.3 Fluorescence as a readout of protease sensor response to protease

**FIGURE 3:**
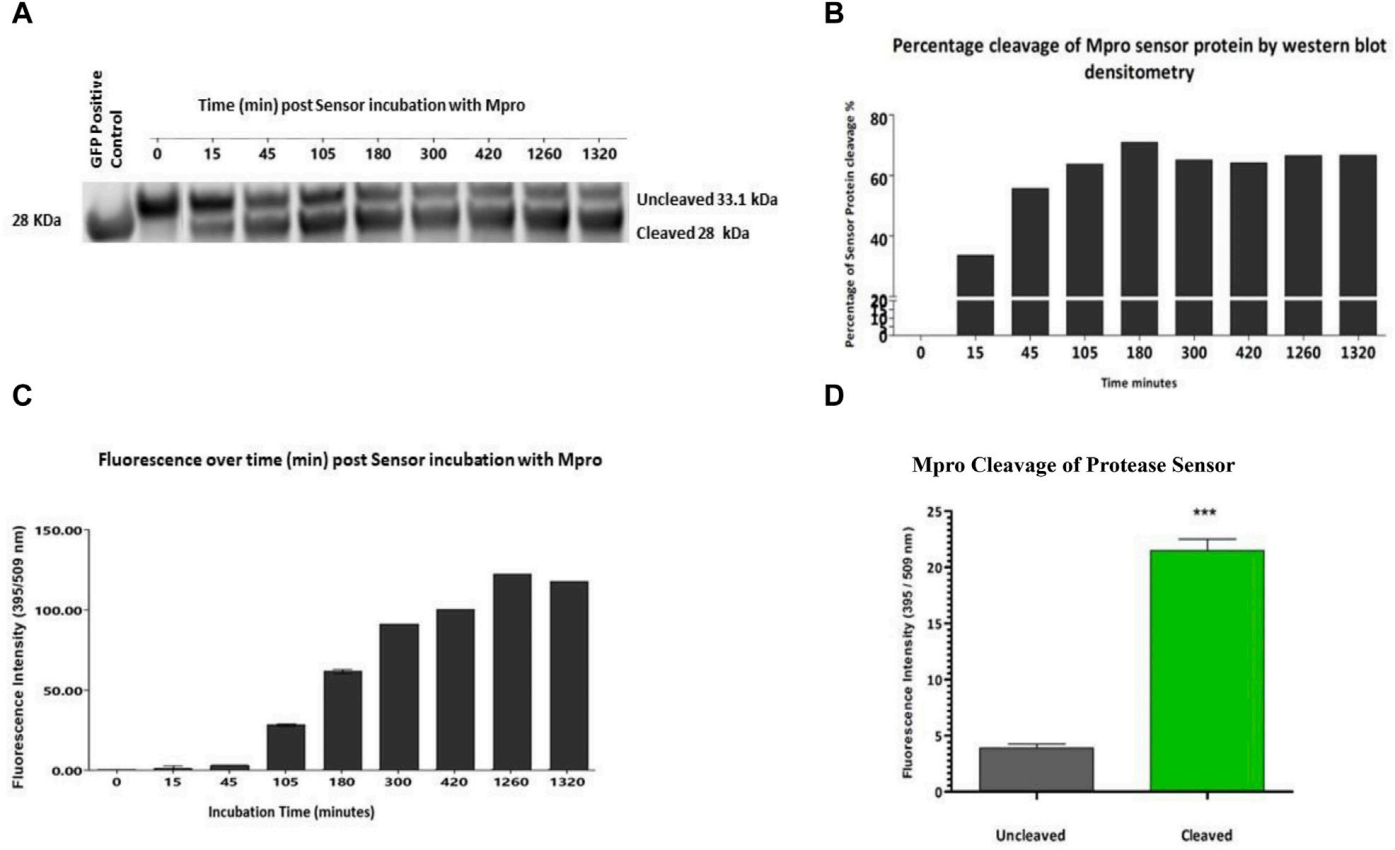
*Fluorescence readout of protease sensor activity All assays have the same components* ± *test protease.* **(A)** *Western blot analysis of cleavage of protease sensor by Mpro protease from 0* min *to 22 h post addition of protease.* **(B)** *Percentage cleavage by densitometry analysis of protease sensor over time.* **(C)** *Gain of fluorescent signal over time, with background uncleaved signal removed. Cleavage precedes the appearance of fluorescence with a ∼ 45 *min *lag (n = 3).* **(D)** *Comparison of the fluorescent signal after 1.5 h incubation of sensor with (grey) or without (green) Mpro protease.*

While not the aim of this study, fluorescence was used as an alternative rapid method of cleavage validation. Once specific cleavage was shown for each construct, fluorescence as a readout was examined. Having previously shown specificity of cleavage and lack of cross-reactivity of the sensor versions, one exemplar construct was chosen to proceed. The Mpro sensor was chosen as it showed the most efficient cleavage ratio by densitometry. The Mpro protease sensor was incubated with Mpro protease with samples of the cleavage assay and fluorescence readings taken over a 24 h period. Within less than 15 min, ∼20% of the sensor was cleaved as measured by densitometry (Figure 3A). Figure 3B shows the percentage of the protease sensor cleaved over time as evidenced by Western blot densitometry. There was a delay in fluorescent signal which we hypothesise to be associated with maturation of the cleaved sensor between 15 min and 45 min, consistent with the known maturation for GFP S65T of 27 min *in vitro* (Figure 3C) (Iizuka et al., 2011). Cleavage of the M2 quencher is followed by fluorophore maturation, possibly contributing to the apparent delay between cleavage signal and fluorescence with cleavage dynamics for each protease also a contributory factor. Complete cleavage was not apparent by 24 h and was found to be the case with all three sensor versions and hypothesised to be a consequence of M2 tetramerisation whereby access to the cleavage site is obstructed in a sterically hindered manner. A significant (*p* < 0.001) gain of fluorescent signal was detected on cleavage, corresponding to 5.5 times the background fluorescence of the sensor in *vitro* assays after 1.5 h incubation with Mpro (Figure 3D).

### 3.4 Immunoassay detection of tag uncoupling

**FIGURE 4:**
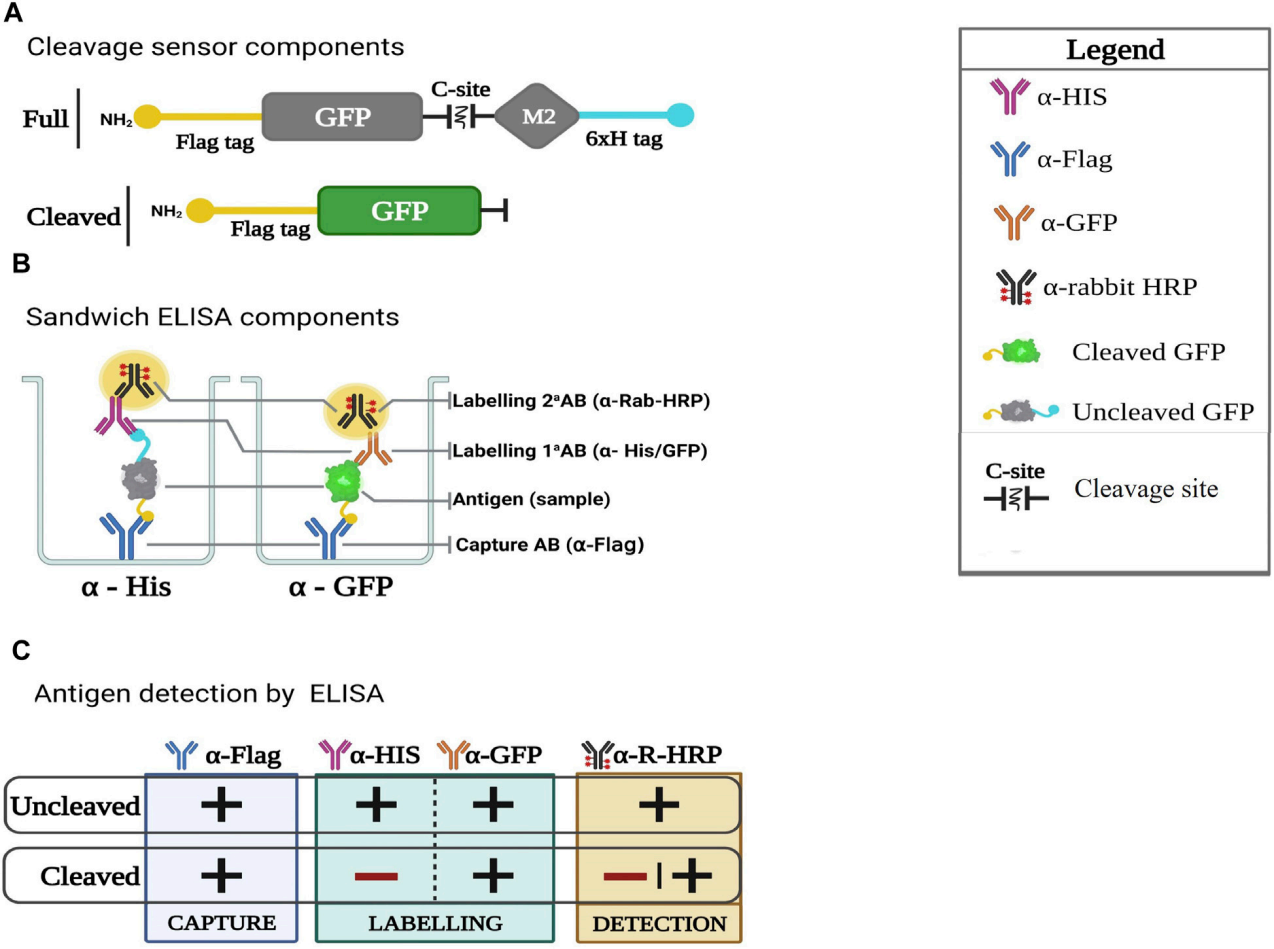
*Immunoassay components* **(A)** Protease sensor diagram comparing full sensor to sensor post cleavage with HIS tagged removed **(B)** *Sandwich ELISA components.* Antibodies used for the reactions: α-HIS antibody detects the full sensor and α-GFP detects both cleaved and uncleaved sensors. **(C)** *Antigen detection by ELISA.* Illustration of sensor detection (full or cleaved with α- HIS or GFP). Cleaved sensors would only be detected with α-GFP and not α-HIS.

The sandwich ELISA-based readout detects the synthetic tags on either side of the protease cleavage module, providing a quantitative readout of uncleaved (dual tag-positive) vs. cleaved (single tag-positive) device. Cleavage detection involves two separate Sandwich ELISAs using already well characterised antibodies, both of which use α-Flag antibody as the capture antibody. Labelling primary antibody differs between the two ELISAs, whereby α-HIS is used for the uncleaved sensor and α-GFP for both cleaved and uncleaved sensors. The secondary labelling antibody is α-rabbit conjugated to horseradish peroxidase (HRP) in order to produce a chemiluminescent signal as a readout. The cleavage site for each sensor is located post the GFP module (Figure 4A), where, upon cleavage, the HIS tag will be separated from the Flag-tagged GFP. Protease sensors cleaved in the presence of their specific protease will show a reduced HIS ELISA signal when compared with uncleaved. Figure 4B illustrates the sandwich ELISA components, with Figure 4C depicting scenarios providing detectable signals.

**FIGURE 5:**
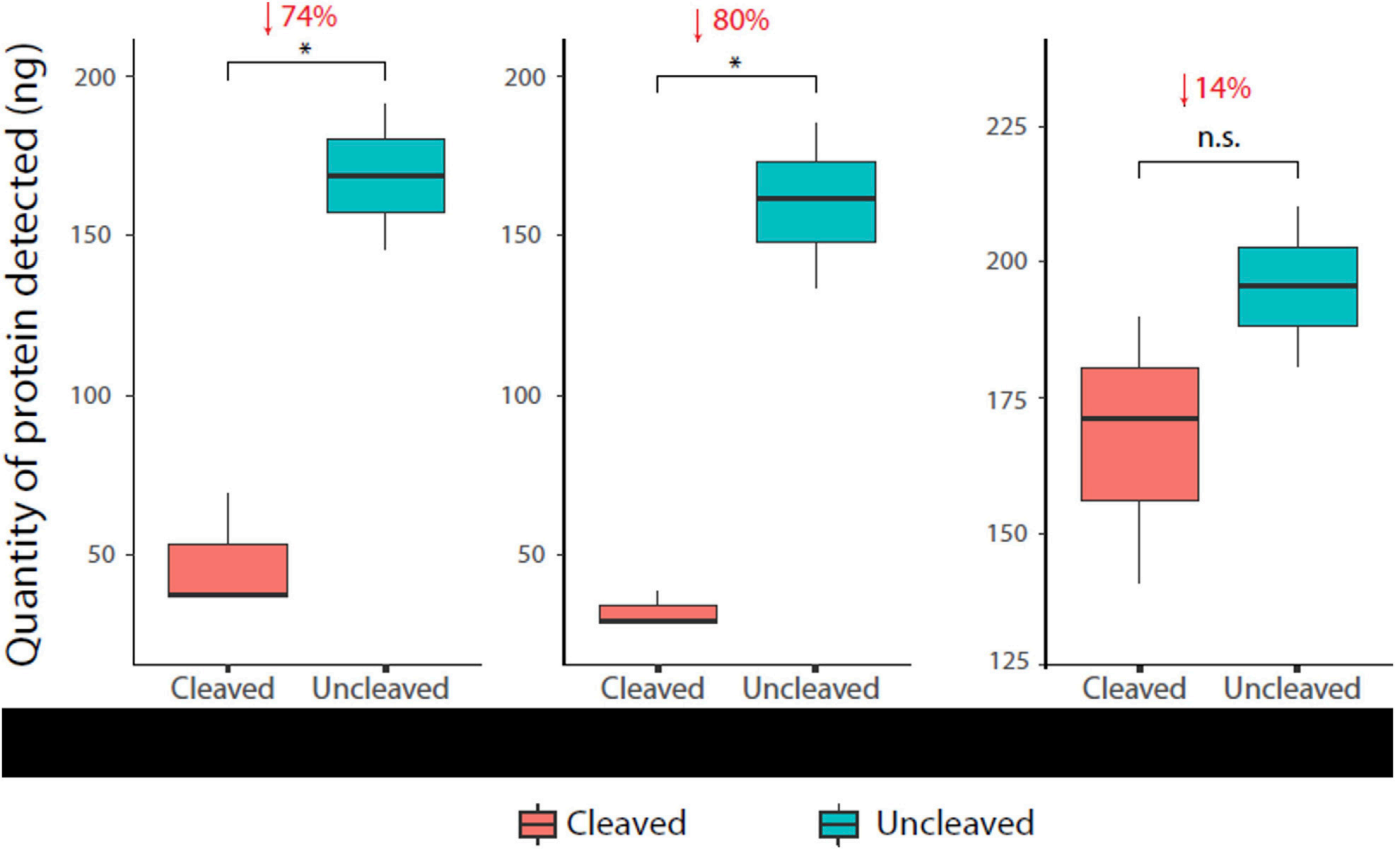
Immunoassay readout of various protease sensors Cleaved (HIS-Flag+) and uncleaved (HIS + Flag+) protein was quantified by the immunoassay ± test protease for each sensor construct. The assay successfully detected cleavage (reduction in α-HIS signal) in all cases, with cleavage patterns matching Western blot analysis (Figure 4).

A reduction in the α-HIS signal of cleaved protease sensors was detected and quantified for each of the three sensor versions. Numbers reported are percentage reduction in protein quantities after normalisation to uncleaved controls, i.e., uncleaved control as 0% reduction. Mpro and TEV versions displayed statistically significant signal reduction of 74% (*p* = 0.01039) and 80% (*p* = 0.0205) respectively. The MMP9 version showed a signal reduction of 14%, although not statistically significant (*p* = 0.2719) (Figure 5). The ELISA proved to be capable of detecting protein quantities as low as 10 ng, as shown in each of the standard curves (Supplementary Figure S1).

## 4 Discussion

As all life on the planet is protein based, proteases are universally distributed throughout all living organisms. Their activity is specific and, as a result, can consequently be detected precisely. This work combines the sequence specificity of any given protease with the simplicity of protein production and the sensitivity of a sandwich ELISA in a novel and straightforward fashion, using already well-characterised antibodies. Protease activity is detected by discriminating between signal reductions in decoupled cleaved dual-tagged positive devices relative to uncleaved.

A synthetic biology approach was used to design the DNA constructs necessary to recombine a protease responsive sensor element separating two peptide tags. This study has shown its adaptability by using 3 disparate proteases, 2 viral and 1 human, associated with 3 different types of disease–a plant pathogen, human cancer, and COVID-19. The results were validated with 3 different readouts: Western blot, fluorescence, and a purpose-designed sandwich ELISA.

While Western blot densitometry demonstrated approximate meaningful differences between cleavage ratios, the ELISA, which was performed in triplicate and measured against individual standard curves for each sensor, revealed cleavage ratios in a more sensitive and accurate manner. Densitometry is useful for approximating and comparing relative amounts of protein, whereas an ELISA is far more sensitive and quantitatively accurate (Oh et al., 2017; Butler et al., 2019). Limits of detection (LOD) were determined to be 20 ng for the α-HIS and 8 ng for the α-GFP (Supplementary Figure S1).

Warren *et al.* described a sandwich ELISA similar to that described in this study, albeit using different capture antibodies, also to detect protease activity (Warren et al., 2014). A crucial difference is the fact that the peptide protease substrate was conjugated to a synthetic iron oxide “nanoworm” with additional synthetic elements including a non-degradable spacer composed of D-isomer amino acids, meaning that this type of reporter cannot be genetically encoded. The fact that the protease sensor in our study can be produced in *E. coli* makes production simple and cost effective.

The assays and components employed in this study to validate the strategy provide proof of concept for the applicability of such an approach. This study employed ‘industry-standard’ well-described tags and matched antibodies. Other tag-antibody combinations could readily be employed. 6xHIS is immunogenic and while suitable for *ex vivo* assays as described here, could be replaced by alternative tags and matched antibodies for *in vivo* application.

Future iterations might better avoid the fluorescent element with a quencher examined here to facilitate easier protein purification and increased cleavage ratio. Indeed, we encountered some difficulties due to the M2 quencher, combined with a high copy number plasmid resulting in accumulation in inclusion bodies necessitating time consuming denaturing purification steps. The M2 quencher also reduced cleavage ratios because of the tetramerisation of the protease sensor. In the future, these elements would be replaced with neutral non-functional peptide spacers of optimum length for cleavage discernment and antibody detection.

Recent Ebola, Zika and COVID-19 epidemics have illustrated the need to develop cost-effective rapid diagnostics (Kelly et al., 2022). Diseases which are idiopathic in nature, e.g., Lyme disease, are generally difficult to diagnose and lead to avoidable complications if left undiagnosed (Rogalska et al., 2021). Orphan diseases, which affect no more than 0.05% of the population (Schaaf et al., 2020) and where there is less commercial incentive to develop diagnostics, are another area where synthetic biology approaches could offer the potential for innovative solutions.

The pervasiveness of proteases in living organisms means that a device based on this principle could be universally applied to detect protease activity across all domains of life. Once the cleavage sequence is known, this platform is adaptable for any given protease. The specificity of proteases, by their very nature of amino acid sequence recognition, endows this platform with the same level of precision for any given particular protease. Any protease associated with a disease state, to be deployed in this type of protease responsive synthetic protein technology, would need to be approached individually, taking into account its level of specificity and any overlap with other proteases in its testing environment.

As time goes by more efficient and economical methods of mapping protease specificity are being developed (Zhou et al., 2020; Ratnikov et al., 2021). Armed with this increasing knowledge index of a given proteases proclivity for its substrate profile, these factors can be taken into account and when used in conjunction with an array of tests for a specific disease can lend credence to a more accurate diagnosis with higher confidence.

Finally, the platform’s simplicity using pre-existing, well-characterised antibodies make this sensor device highly applicable. This novel method could be could potentially be applied to any biological liquid sample, such as blood or saliva for detection of protease activity to reveal pathogens, disease biomarkers, *etc.* It could also be applied in environmental settings such as waste water from industry, hospitals or homes to detect environmental biological contaminants. Development as an at-home test means it could be used as a stand-alone or as a prelude to other types of testing. Further development and optimisation are required to produce a rapid, cost-effective and easy-to-operate test. Still, as outlined in this work, it is within the bounds of current technologies. Large-scale screening, with high population coverage and early diagnosis, is critical to the commencement of appropriate treatment or intervention with the ability to do this outside of the laboratory setting, at home or in developing countries where resources for healthcare are limited, there is the potential to save many lives.

## Data Availability

The original contributions presented in the study are included in the article/Supplementary Material, further inquiries can be directed to the corresponding authors.
