# Supplementary material for: Synthetic protein protease sensor platform
Source: Front Bioeng Biotechnol. 2024 Apr 5;12:1347953. doi: 10.3389/fbioe.2024.1347953 (PMC11026627; doi:10.3389/fbioe.2024.1347953)
Supplement: Supplementary file 1 [file DataSheet1.docx]

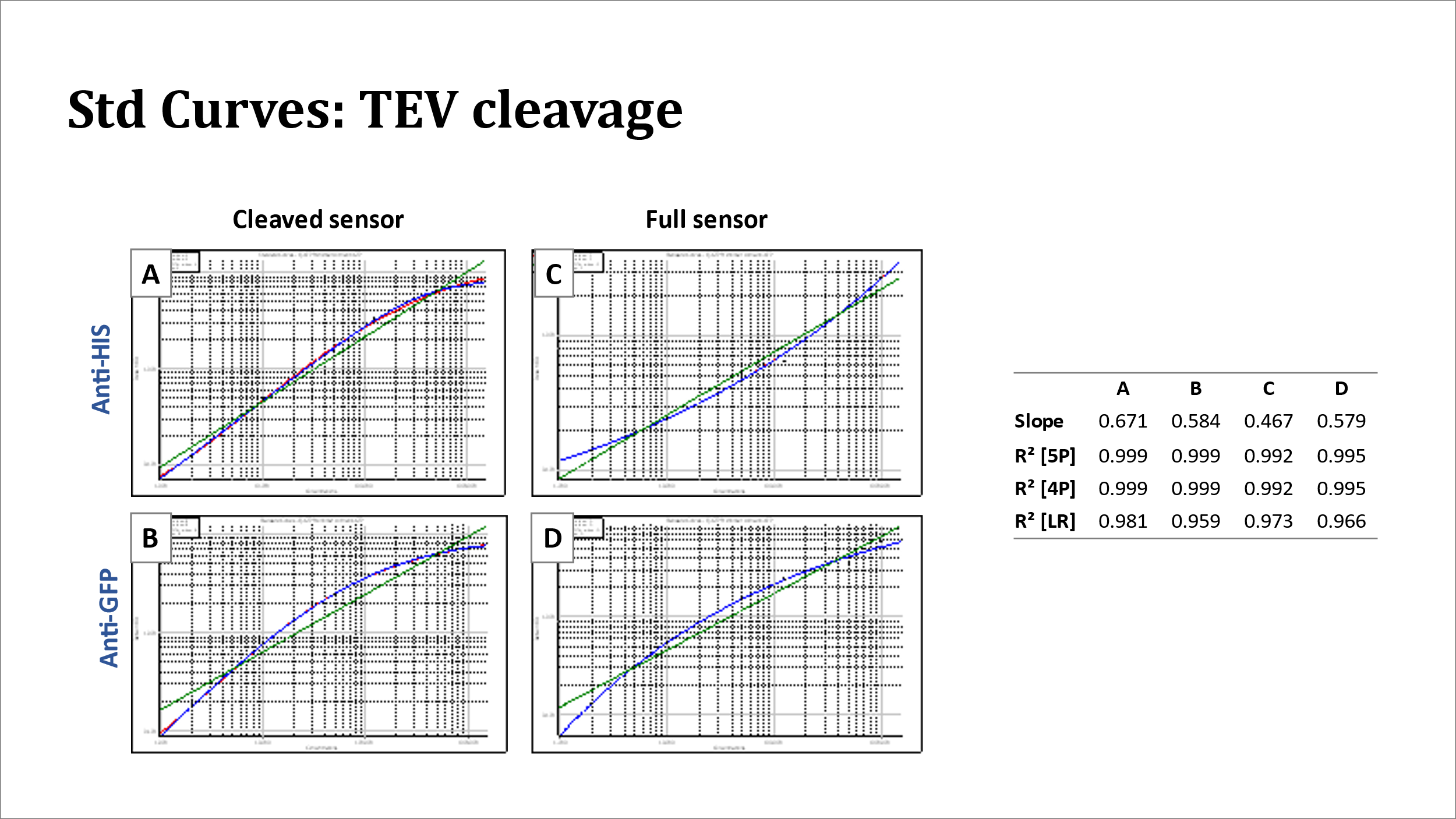


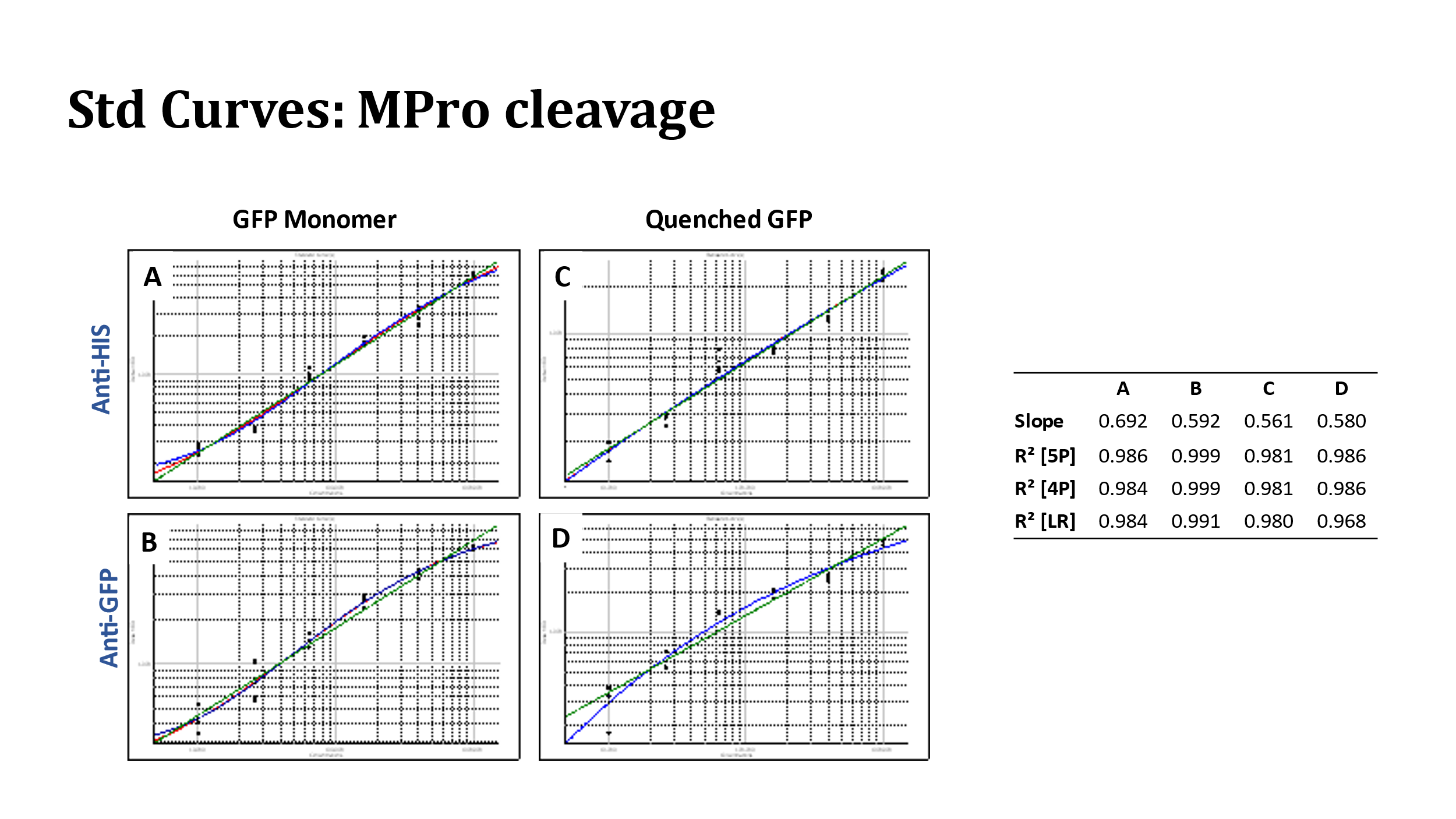


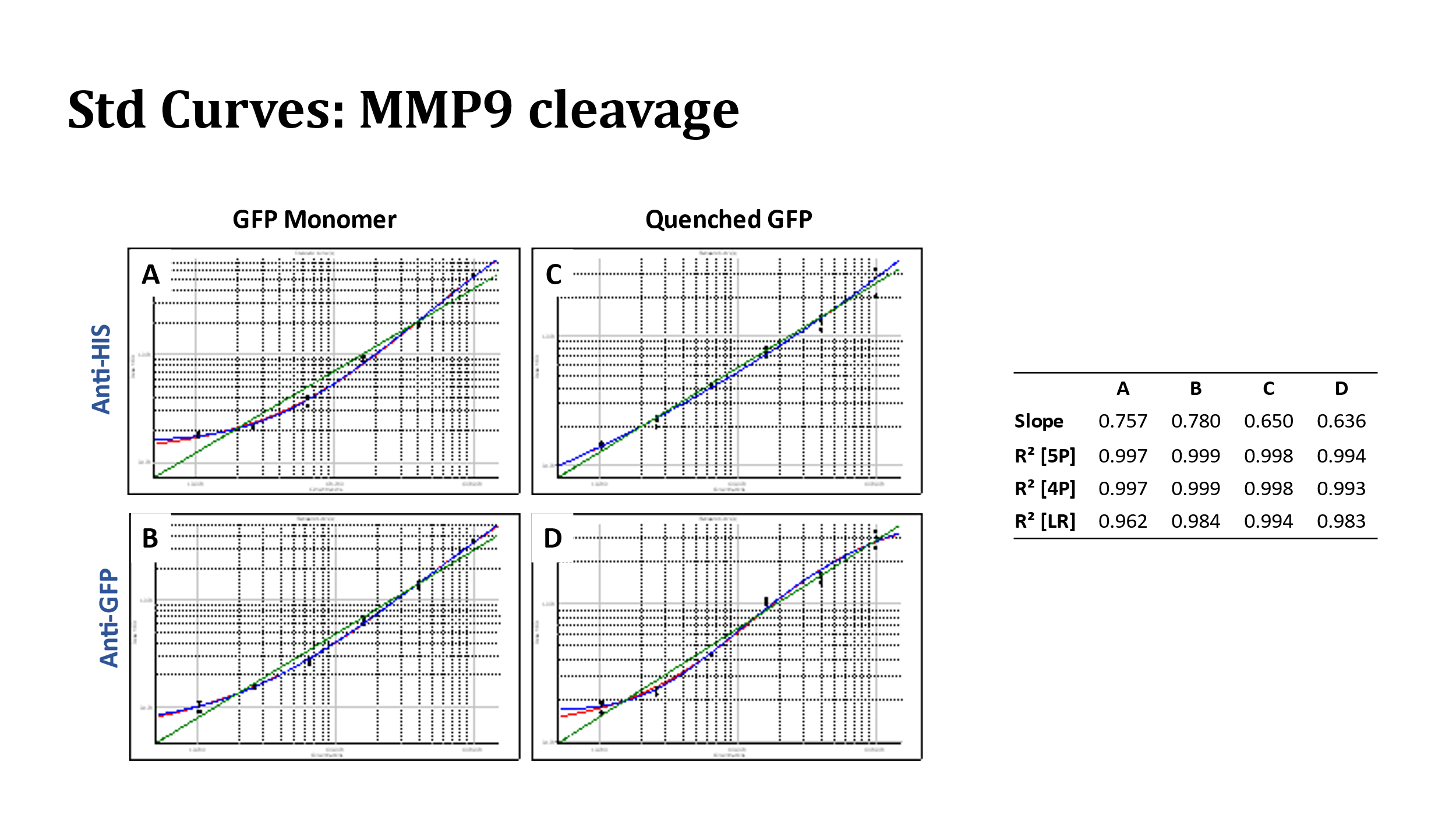


**Supplementary Figure 1. Example of standard curves analysed.** Four standard curves were plotted for each ELISA plate analysing data for each protease. Here are the standard curves for sensors cleaved with TEV protease and the R^2^ values for each. In blue: 5PL-fit, in red: 4PL-fit and in green: linear regression (LR). 5P-fit was chosen to account for differences in symmetries. The plots were extracted as raw data from BMG Omega – data analysis software.


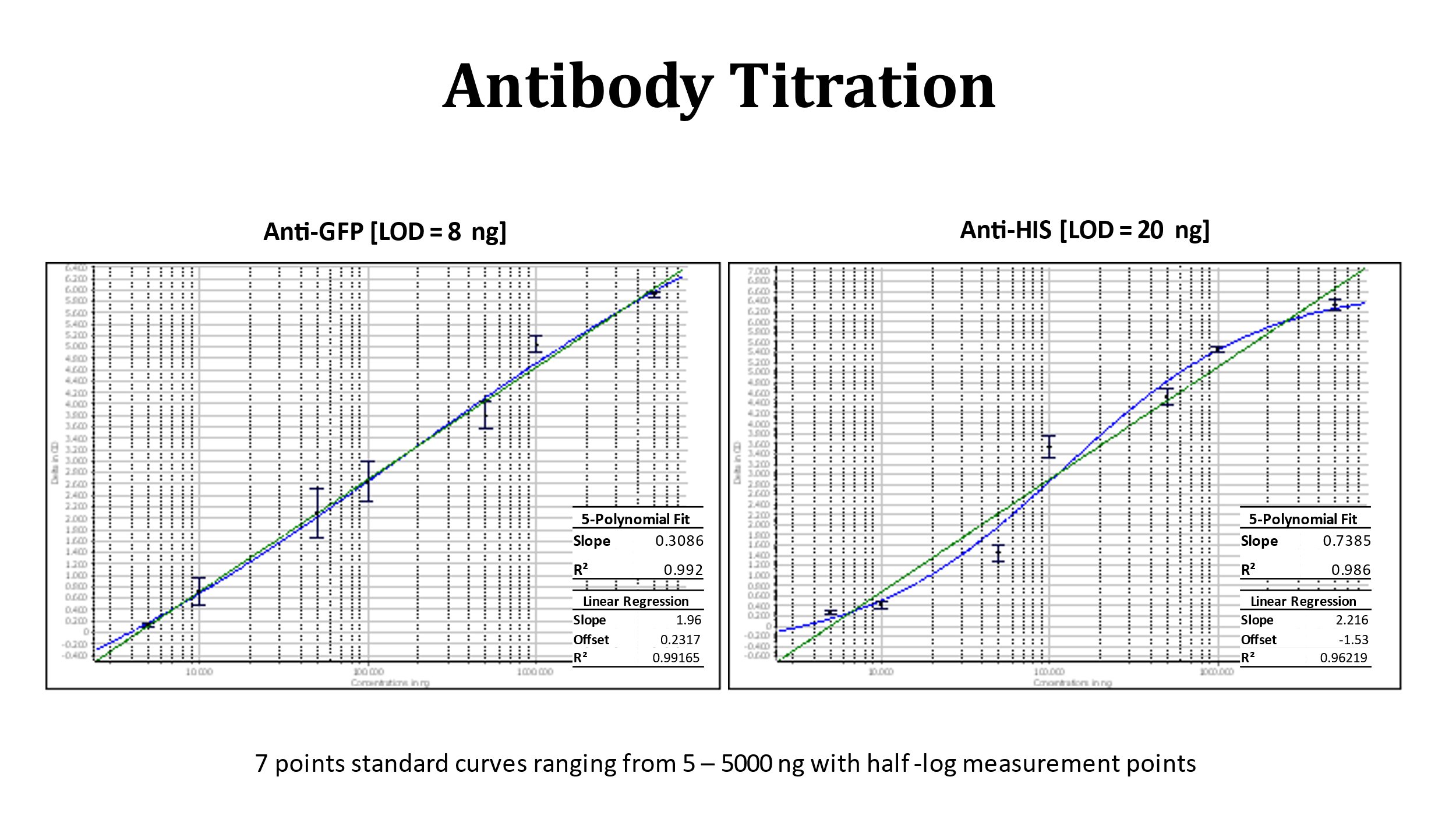


**Table 1**. Antibodies used for quantitative sandwich ELISA

| Antibody | Host | Product No. | Quantity used (per well) |
| --- | --- | --- | --- |
| α-Flag | Goat | ab95045 | 100.00 ng |
| α-HIS | Rabbit | ab9108 | 20.00 ng |
| α-GFP | Rabbit | ab290 | 8.00 ng |
| α-Rabbit HRP | Donkey | ab7083 | 8.00 ng |

**Table 2**. Reagents used for quantitative sandwich ELISA

| Reagent | Recipe |
| --- | --- |
| Phosphate Buffer Saline (PBS) | 1X dilution of a 10X PBS stock (Sigma-Aldrich, DE, Cat. No. P7059) |
| Blocking Solution | 1% w/v of BSA (Sigma-Aldrich, A3294) filtered sterilised (0.2 µm filter) |
| Wash buffer (PBS-T) | 1X PBS + 0.05% v/v of Tween-20 (Sigma-Aldrich, P1379) |
| Elution buffer (EB) | 0.2% w/v of BSA (Sigma-Aldrich, A3294) filtered sterilised (0.2 µm filter) |
